# Three-dimensional building of anisotropic gold nanoparticles under confinement in submicron capsules†

**DOI:** 10.1039/d3na00683b

**Published:** 2023-09-06

**Authors:** Ryuichi Yamada, Makoto Kuwahara, Shota Kuwahara

**Affiliations:** a Department of Chemistry, Faculty of Science, Toho University Funabashi Chiba 274-8510 Japan syouta.kuwahara@sci.toho-u.ac.jp; b Graduate School of Engineering and Institute of Materials and Systems for Sustainability, Nagoya University Chikusa Nagoya 464-8603 Japan

## Abstract

The low collision rate and contact time of gold nanoparticles (NPs) in solution afford a low welding probability, which hinders their welding structure, orientation, and dimension. Encapsulated anisotropic NPs, gold nanotriangles (AuNTs), were successfully assembled into a three-dimensional structure inside a permeable silica nanocapsule under light illumination to generate localized surface plasmon resonance (LSPR). AuNTs were trapped in the permeable silica nanocapsules and diffused in the nanospace because of copolymer release, which increased the contact probability of AuNTs and promoted the three-dimensional building of AuNTs. Electron energy loss mapping simulations revealed that the obtained three-dimensional AuNT structure exhibited spatially separated multiple LSPR modes with different energies of incident light, which are photophysically attractive beyond the facet-selective chemical growth of NPs, and postmodification for anchoring substances with site-selective attachment to the obtained structure will be applicable to expand the sensing design and class of substances for sensing.

## Abstract 2

Encapsulated gold nanotriangles (AuNTs) were assembled into a three-dimensional structure inside a permeable silica nanocapsule under light illumination to generate multiple localized surface plasmon resonance modes with different energies.

## Graphical abstract

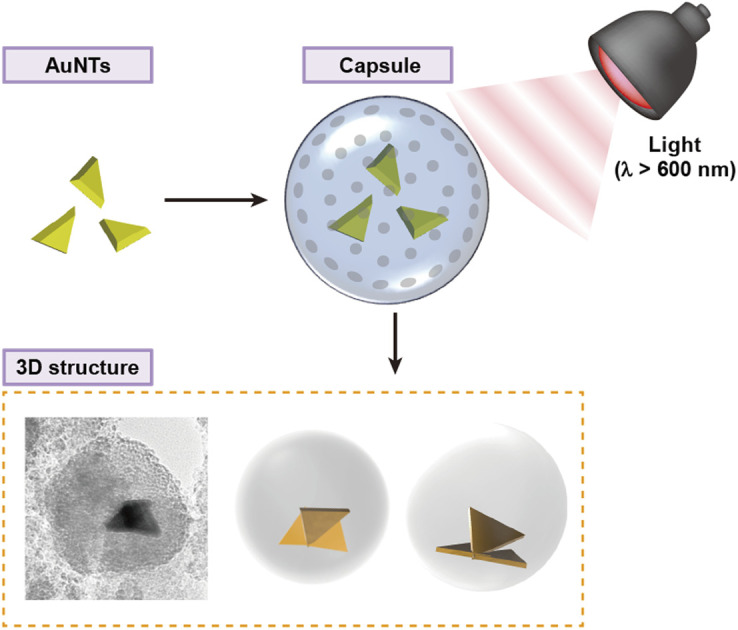

## Introduction

The optical properties of metal nanoparticles (NPs) are characterized by their localized surface plasmon resonance (LSPR), and the resonant energy of LSPR depends on their size, shape, and morphology.^1–3^ Metal NPs have been utilized in sensors,^4–7^ bioimaging,^7–9^ photo-therapy,^10,11^ photocatalysts,^12–14^ and solar cells^15,16^ owing to their unique optical properties related to LSPR. When multiple plasmon modes with different energies are induced in metal NPs, they exhibit multicolor characteristics for plasmonic applications, advancing multicolor sensing and wide-range light harvesting and conversion devices.

The anisotropic structure of NPs generates multiple plasmon modes according to different resonant energies in each anisotropic direction.^2,6,17–19^ On the other hand, the LSPR characteristics of NPs can also be modulated by building an assembly of NPs because of the change in the individual plasmonic structure and/or generation of coupled LSPR modes in the gap region.^20–23^ Assembled structures of NPs can be controllably fabricated *via* a bottom-up approach, such as DNA nanotechnology,^24–26^ or a top-down approach, such as lithography of a metal-evaporated layer on a well-aligned nanostructure.^27–30^ Plasmonic superparticles with three-dimensional structures obtained by chemical synthesis *via* thiol-ligand-mediated growth,^31^ surface-strain-modulated seeded growth^32^ and self-assembly with solvophobic interactions between NPs and a solvent^33^ show broadband light absorption, and are included in many promising applications such as solar energy conversion and photothermal therapy.

Recently, our group reported the site-selective welding of adjacent gold nanotriangles (AuNTs) under continuous light illumination and the obtained electromagnetically continuous structure successfully exhibited a new plasmon mode.^17^ Incident light-induced LSPR in AuNTs generates plasmonically enhanced hot electrons, subsequently causing surface diffusion and migration of Au atoms in the contact region of the AuNTs.^17,34,35^ The proposed mechanism of NP welding indicates that this technique can be used to attach adjacent NPs depending on their induced LSPR, realizing controllable nanostructure building using NPs. However, the collision rate and contact time of NPs in solution afford a low welding probability *via* the light-induced LSPR; the assembled film of NPs restricts the welding structure and orientation to a two-dimensional prearranged alignment.

Herein, the light-induced welding of NPs in a confined submicron space is proposed to increase the collision rate and assemble a fixed number of NPs in advance. For this purpose, a confined submicron space with the following advantages for NP welding is required: (1) easy handling and controlling of the NP confinement, (2) submicron hollow space for free movement of NPs, and (3) presence of a seamless and chemically stable shell. Recently, Liz-Marzán and coworkers synthesized NP clusters in permeable silica nanocapsules^36^ and realized cyclic aggregation and reproducible plasmon band shifts at different solvent compositions. In the synthesis of silica nanocapsules, the AuNPs clusters encapsulate within a block-copolymer micelle, then the resulting polymeric micelles are coated with mesoporous silica obtained by the hydrolysis of tetraethyl orthosilicate. Subsequently, the encapsulated copolymer is released outside of the capsule, and AuNPs are moved freely inside the silica nanocapsules. The NP-confined nanocapsules satisfied the required conditions for the construction of high-yield three-dimensional welded NPs as mentioned above. Herein, hydrophobic capping agent-free AuNTs confined in permeable silica nanocapsules were synthesized, following the connection of AuNTs *via* LSPR-induced welding under continuous light illumination to afford a three-dimensional NP-based structure.

## Results and discussion

### LSPR induced welding of AuNTs in a solution

**Fig. 1:**
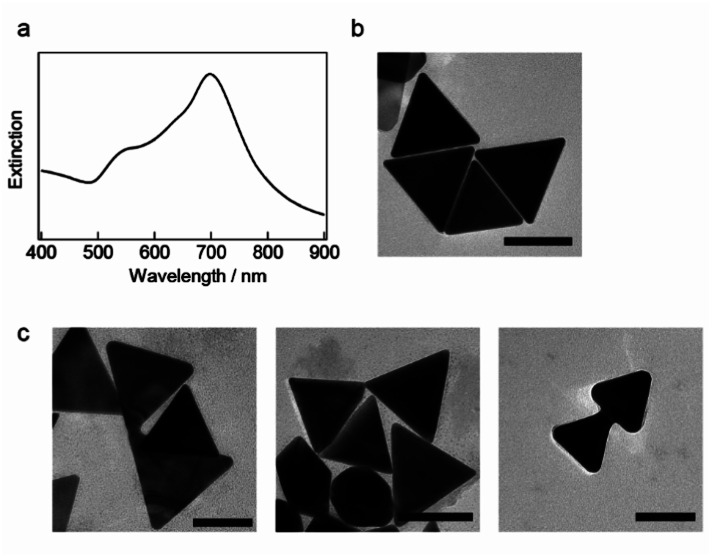
(a) Optical extinction spectrum and (b) transmission electron microscopy (TEM) image of the synthesized gold nanotriangles (AuNTs) (scale bar: 100 nm). (c) TEM images of welded AuNTs obtained after light illumination (*λ* > 600 nm) for 1 h. The scale bar is 100 nm.

The seedless growth method based on the study by Chen and coworkers was used to synthesize AuNTs, which were then encapsulated in polymeric micelles and coated with a silica shell.^37^ The optical absorption spectrum of the synthesized AuNTs exhibits an extinction band at ∼698 nm and a weak shoulder at ∼560 nm (Fig. 1a), which correspond to the characteristic LSPR of AuNTs. Transmission electron microscopy (TEM) images in Fig. 1b show that AuNTs with sharp tips were successfully prepared and the average edge length of the obtained nanoplates was 118 ± 11 nm. The yield of AuNTs was 70%, and spherical and polygonal gold NPs were observed in the obtained AuNTs sample.

As previously reported,^17^ continuous light with a cut-on wavelength of 600 nm (*λ* > 600 nm) was used to illuminate the AuNTs in a microtube to weld AuNTs by selectively generating the plasmon modes in the corner of the AuNTs. Light-illuminated AuNTs were studied *via* TEM, as shown in Fig. 1c and S2,† and the TEM images show the two adjacent AuNTs welded at the hot spot as the simulated localized electromagnetic field distribution by Sun and coworkers,^18^ which formed in standing side by side (left), facing each corner (middle) and aligning two AuNTs by facing one corner and the other edge (right). The welding AuNTs in Fig. 1c supports the previously proposed the LSPR-induced welding mechanism wherein hot electrons plasmonically enhanced using an incident electromagnetic field subsequently induce surface diffusion and migration of Au atoms in the contact region of the AuNTs. However, only ∼5% of AuNTs are joined during the welding process because of the low contact probability of AuNTs in a solution. Then, the light-induced welding of NPs in a confined submicron space to increase the collision rate and build a three-dimensional structure with anisotropic NPs was studied.

### Properties of permeable silica capsules containing gold NPs

**Fig. 2:**
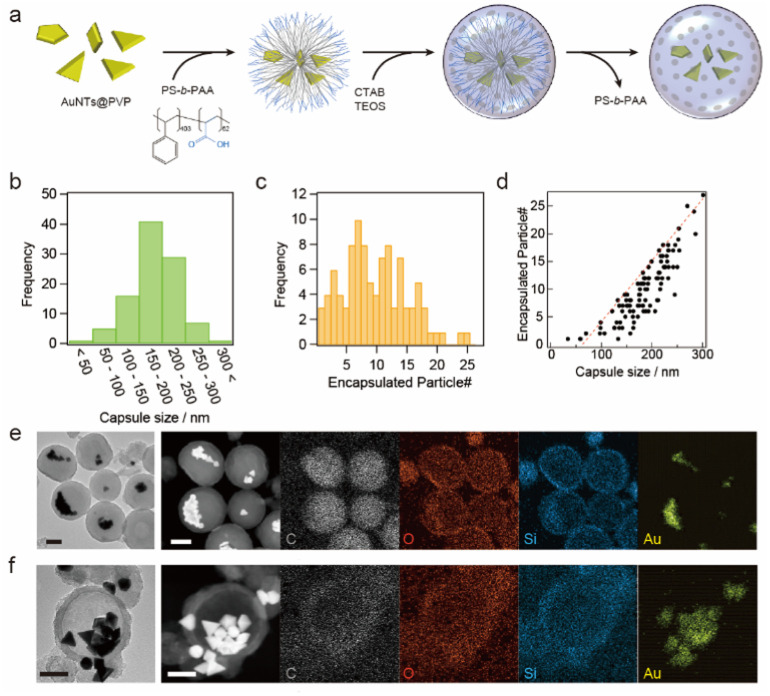
(a) Schematic of the fabrication of permeable silica capsules containing gold NPs. (b and c) Histograms showing the distribution of the (b) size of silica nanocapsules and (c) number of encapsulated particles per capsule. (d) The number of the encapsulated gold NPs as a function of the size of silica nanocapsules. The red dotted line shows the maximum number of encapsulated NPs against the size of a capsule. (e and f) STEM-EDX elemental analysis of synthesized gold NP-encapsulated silica nanocapsules (e) before and (f) after block copolymer release. Scale bars are 100 nm.

The synthesized AuNTs were confined in permeable silica nanocapsules, as shown in Fig. 2a. The obtained nanocapsules were studied *via* scanning electron microscopy (SEM) and TEM, and the images shows that AuNTs and spherical and polygonal gold NPs were encapsulated inside the silica nanocapsules (Fig. S3†). The diameter of the nanocapsules was 185 ± 48.5 nm, and they contained 10 ± 6 gold NPs (Fig. 2b and c). The number of NPs encapsulated in the nanocapsules increased with an increase in the nanocapsule diameter, as shown in Fig. 2d, and the maximum number of encapsulated NPs was proportional to 0.11 × (the diameter of nanocapsules) − 6.94, indicating that with a 10 nm increase in the nanocapsule diameter, the nanocapsule could accept one additional NP inside. While hydrophobic capping agent-free AuNTs were used for the synthesis of AuNTs confined in silica nanocapsules, unlike previous report,^36^ AuNTs aggregated when the PVP-stabilized AuNTs were added in the tetrahydrofuran (THF)/water mixture or a poly(styrene-*b*-acrylic acid) (PS-*b*-PAA) solution was added in the THF/water mixture solution and subsequent polymer capsule formation proceeds according to the AuNT aggregate size.

The copolymer release from the silica nanocapsules was confirmed *via* scanning TEM-energy-dispersive X-ray spectroscopy (STEM-EDX) elemental analysis, which also visualized the changes in carbon intensity of the copolymer^36^ as carbon is the main constituent of copolymers. Fig. 2e and f show that the carbon distribution in the silica nanocapsules was observed before copolymer release whereas no carbon distribution was observed after copolymer release.

Furthermore, the distributions of other elements (Si, O, and Au) remained unchanged after the copolymer release process, indicating that the formed permeable silica shell was stable before and after polymer release. Note that the representative TEM image after the copolymer release process (Fig. S4†) indicates low contrast inside the silica shell, which also supports the encapsulated constituent, copolymer, was released to the outside of the silica nanocapsules.

### Structure and predicted LSPR property of three-dimensional NP-based structure

**Fig. 3:**
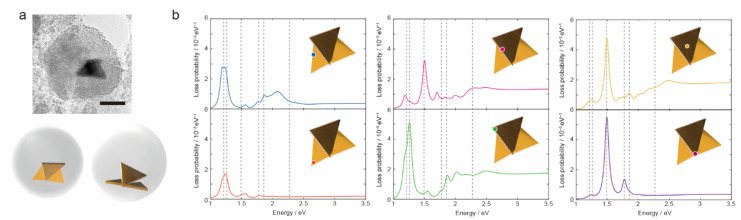
(a) TEM image of AuNTs encapsulated in silica nanocapsules under light illumination and proposed model structures of the welded three AuNTs obtained from the TEM image. Scale bar is 50 nm. (b) Simulated electron energy loss (EEL) spectra from the edge (blue) and corner (red) of the two welded AuNTs on the *xy* plane, the edge (pink), corner (green), face (yellow) of the standing AuNT along *z* axis, the welded three AuNTs point (purple) obtained by the proposed welded gold nanostructure in (a). The welding two AuNTs was placed on the *xy* plane, and observed from *z*-axis. The black dotted lines indicate the energies of 1.20, 1.26, 1.49, 1.77, 1.86 and 2.27 eV.

The AuNTs encapsulated in silica nanocapsules were illuminated by continuous light with a cut-on wavelength of 600 nm (*λ* > 600 nm) for 10 min to building three-dimensional NP structures *via* the LSPR-triggered welding process. The TEM image in Fig. 3a shows that three AuNTs construct the three-dimensional structure inside the silica nanocapsule. Electron energy loss (EEL) spectrum and mapping were performed *via* the boundary element method calculation using the NMPBEM toolbox, Matlab toolbox for the simulation of metallic nanoparticles using a boundary element method approach^38^ on the proposed structure of the three welded AuNTs obtained from the TEM contrast. Fig. 3b shows simulated EEL spectra from the six spots on the proposed welding gold nanostructure shown in Fig. 3a. In the range of 1–1.3 eV, the high intensity of loss probability in EEL spectra appeared at the edge and corner of the three AuNTs in the three-dimensional NP-welded structure. Then, the loss probability with high intensity was shown in the welded three AuNTs point at the energy of ∼1.5 eV. As increased in the energy of incident electrons (>2 eV), the standing AuNTs along the *z* axis had higher loss probability than the AuNTs on the *xy* plane. The EEL spectra indicated that different plasmon modes with different energies were generated on the three AuNTs in the three-dimensional NP-welded structure and that the intensities of loss probability of each LSPR mode depended on spots on the structure.

**Fig. 4:**
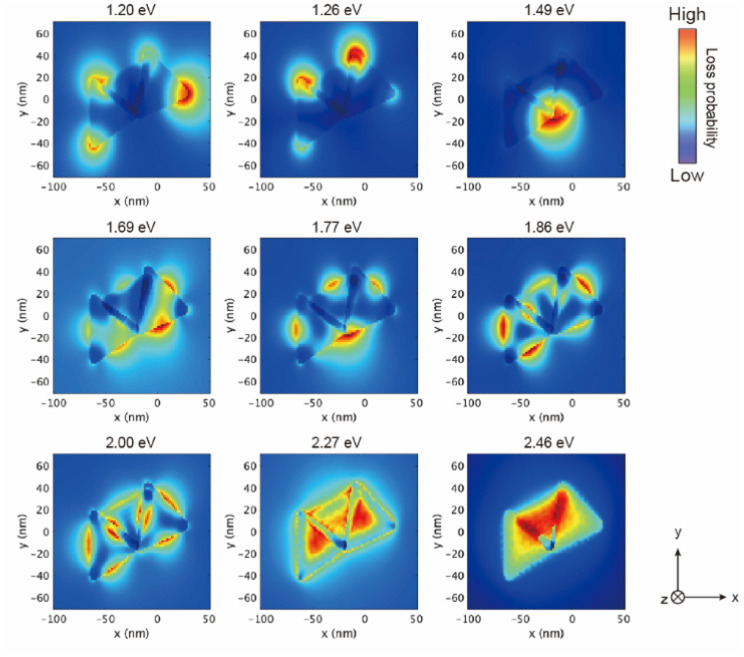
Simulated electron energy loss (EEL) maps of the proposed welded gold nanostructure in (a). The welding two AuNTs was placed on the *xy* plane, and observed from *z*-axis.

The EEL maps shown in Fig. 4 indicate that the plasmon mode generate with an energy of 1.20 eV localized at the corner of the two welded AuNTs on the *xy* plane, then EEL spots with high intensity changed to the three AuNTs welding part (1.49 eV); the edge of the two welded AuNTs on the *xy* plane (1.69–1.77 eV). In the range from 1.86 to 2.46 eV, the standing AuNT along the *z* axis was involved in the plasmon mode generated in the three-dimensional structure: the edge spread over the three welding AuNTs (1.86 and 2.00 eV); the face of the standing AuNT along the *z* axis (2.27 and 2.46 eV). The obtained three-dimensional NP-welded structure generates multiple LSPR modes at spatially separated positions on the structure.

**Fig. 5:**
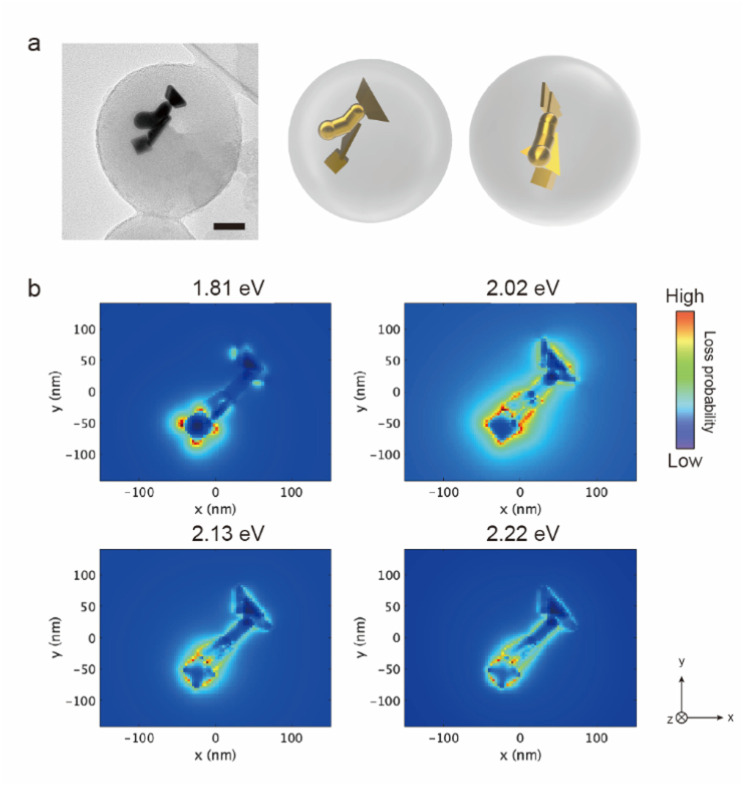
(a) TEM image of gold NPs encapsulated in a silica nanocapsule under light illumination and the proposed model structures of the welded three AuNTs observed in the TEM image. Scale bar is 50 nm. (b) EEL maps of the welded gold nanostructure in (a).

Since AuNTs were trapped in the permeable silica nanocapsules and moved around the nanospace because of the copolymer release, the contact probability between AuNTs increased to more than 70%, which promotes the building of three-dimensional NP structures. However, thermal melting also caused gold NP welding caused by the temperature increase by the encapsulated gold NPs within a tiny space,^20^ whereas thermal equilibrium in the solution of gold NPs caused the position-selective welding induced by LSPR (Fig. 5a). The obtained three-dimensional NP-welded structure also generates multiple LSPR modes at spatially separated positions on the structure as well as the three welded AuNTs (Fig. 5b). All observable silica nanocapsules by TEM contained three-dimensional NP-welded structures inside the nanocapsules after the light illumination although overlapped NPs made the welding structures difficult to be characterized. The optimization of light-illuminated conditions, such as time for welding, intensity, and wavelength, can improve the site selectivity for building three-dimensional structures using gold NPs *via* LSPR-originated welding. Moreover, the silica shell of nanocapsules makes it difficult to acquire three-dimensional structural data and EEL mapping of the obtained gold NP-welded structure, and its removal *via* hydrolysis will simplify structural and optical measurements of the obtained three-dimensional structure of gold NPs and facilitate the application of the structure to sensing.

## Experimental section

### Materials and methods

Methoxypolyethylene glycol silane (silane-PEG, Mw: 10 000) was purchased from Polysciences, Inc. Poly(styrene-*b*-acrylic acid) (PS_403_-*b*-PAA_62_, Mw: 41 000 (PS) and 4200 (PAA)) was purchased from Polymer Source. All the chemicals were used as received.

TEM images were captured using an electron microscope at 120 kV (Tecnai G2 F20 S-TWIN, FEI, Thermo Fisher Scientific, USA). STEM images were captured at 30 kV (SU9000, Hitachi, Japan). STEM-EDX was performed using an X-Max 80T (Oxford Instruments, UK). Optical absorption spectra of the obtained samples were collected using an ultraviolet-visible-near infrared (UV-vis-NIR) spectrophotometer (UV-3600, SHIMADZU Corp., Japan).

### Synthesis of AuNTs

1.6 mL of aqueous 0.1 M hexadecyltrimethylammonium chloride (CTAC, Tokyo Chemical Industry Co., Ltd, Japan) was added to 8.0 mL of Milli-Q water. Then, 75 μL of a 0.01 M KI solution (FUJIFILM Wako Pure Chemical Corp., Japan) and 80 μL of an aqueous 25.4 mM hydrogen tetrachloroaurate trihydrate solution (HAuCl_4_·3H_2_O, Merck, Sigma-Aldrich, USA) were sequentially added to the CTAC solution. The obtained yellow solution was changed to a colorless solution by adding 20.3 μL of a 0.1 M NaOH solution (FUJIFILM Wako Pure Chemical Corp., Japan) and 80 μL of a 0.064 M l-ascorbic acid solution (FUJIFILM Wako Pure Chemical Corp., Japan) sequentially. Finally, 10 μL of 0.1 M NaOH was injected into the solution, and the vial containing the mixed solution was vigorously shaken for 3 s. The shaken solution was kept at room temperature for 10 min to grow AuNTs. After the growth of AuNTs, 2 mL of the solution was centrifuged at 15 000*g* for 10 min and the supernatant was removed. The obtained sediment was redispersed in 100 μL of 0.1 M CTAC and 1.8 mL of Milli-Q water; then, 0.2 mL of an aqueous 10 mg mL^−1^ polyvinylpyrrolidone solution (PVP, Tokyo Chemical Industry Co., Ltd, Japan) was added and stirred for 24 h, affording AuNTs@PVP.

### Preparation of permeable silica capsules containing gold NPs

AuNTs@PVP was centrifuged at 15 000*g* for 10 min, and the supernatant was removed. The sediment was redispersed in a mixed solution of 2 mL of Milli-Q water and 8 mL of THF (Nacalai Tesque Inc., Japan). A solution of PS_403_-*b*-PAA_62_ in THF (2 mL, 6 mg mL^−1^) was added dropwise to the dispersion under magnetic stirring. Subsequently, the water content in the solution was increased to 35 wt% by adding Milli-Q water, and the obtained solution was heated at 70 °C for 30 min in a water bath. The obtained solution was centrifuged at 1600*g* for 30 min, and the supernatant was removed. The sediment was washed with water and redispersed in 2 mL of Milli-Q water.

40 μL of an aqueous 1.5 mM CTAB solution was added to the dispersion containing AuNTs@PVP and PS_403_-*b*-PAA_62_, and after incubation for 1 h of the mixed solution, three aliquots of tetraethyl orthosilicate (Merck, Sigma-Aldrich, USA) solution (12 μL, 20 vol% in ethanol) was added to the dispersion at 1 h intervals, followed by stirring for 24 h. The obtained solution was centrifuged at 1600*g* for 30 min, and the supernatant was removed. The sediment was washed with water and redispersed in 2.5 mL of ethanol, and the AuNTs encapsulated in silica nanocapsules were obtained.

A solution of 10 mg mL^−1^ silane-PEG (0.25 mL, 10 mg mL^−1^ in ethanol) in ethanol was added dropwise to the silica-coated polymer capsules under sonication. The mixture was reacted for 12 h at 40 °C with mild stirring. PEG-modified silica-coated clusters were centrifuged at 1600*g* for 30 min, and the sediment was washed with water and redispersed in 0.125 mL of Milli-Q water. The dispersion of silane-PEG-coated polymer capsules was diluted using 5 μL of THF and moderately stirred for 2 h to remove the encapsulated PS_403_-*b*-PAA_62_.

The obtained AuNT-encapsulated polymer capsules were illuminated for 10 min using a xenon lamp (MAX-303, compact xenon light source, Asahi Spectra Co., Ltd, Japan) with a long-pass filter with a cut-on wavelength of 600 nm (45 mW cm^−2^) After illumination, the samples containing AuNT-encapsulated polymer capsules were collected for subsequent TEM analysis.

## Conclusions

Encapsulated anisotropic NPs were assembled into a three-dimensional structure inside a silica nanocapsule under light illumination, which generates multiple LSPR modes with different energies against the incident electromagnetic field at spatially separated positions on the structure. The contact probability between AuNTs was increased by confining them in a nanospace, which promotes the building of three-dimensional gold NP structures. The light illumination process generated LSPR welding for site-selective bonding between gold NPs and thermal melting, which may preserve the obtained three-dimensional gold NP structure. Herein, the multiple LSPR modes with spatially different positions are photophysically attractive beyond the facet-selective chemical growth of NPs, and postmodification for anchoring substances with site-selective attachment to the obtained structure will be applicable to multicolor sensing with vary chemical recognition. Three-dimensional arrangement of target molecules on the NP structure with spatially different multiple LSPR modes will expand the sensing design and class of substances used in sensing applications.

## Author contributions

R. Y. synthesized and characterized the AuNTs encapsulated in silica nanocapsules. M. K. simulated the EEL maps of the welded AuNTs as well as the observation of the nanocapsules by SEM and TEM. R. Y. and S. K. conducted the experiment. S. K. wrote the paper, and M. K. helped to write the paper. All authors reviewed the manuscript.

## Conflicts of interest

There are no conflicts to declare.

## Supplementary Material

NA-005-D3NA00683B-s001
