# Supplementary material for: Three-dimensional building of anisotropic gold nanoparticles under confinement in submicron capsules
Source: Nanoscale Adv. 2023 Sep 6;5(21):5780–5. doi: 10.1039/d3na00683b (PMC10597547; doi:10.1039/d3na00683b)
Supplement: NA-005-D3NA00683B-s001 [file NA-005-D3NA00683B-s001.pdf]

Electronic Supplementary Material (ESI) for Nanoscale Advances.
This journal is © The Royal Society of Chemistry 2023

Supporting Information

Three-Dimensional Building of Anisotropic Gold Nanoparticles under Confinement in Submicron Capsules

Ryuichi Yamada,[†] Makoto Kuwahara,[‡,§] and Shota Kuwahara[*,†]

[†]Department of Chemistry, Faculty of Science, Toho University, 2-2-1 Miyama, Funabashi, Chiba 274-8510, Japan

[‡]Graduate School of Engineering, Nagoya University, Furo-cho, Chikusa, Nagoya 464-8603, Japan

[§]Institute of Materials and Systems for Sustainability and Institute of Materials and Systems for Sustainability, Nagoya University, Furo-cho, Chikusa, Nagoya 464-8603, Japan

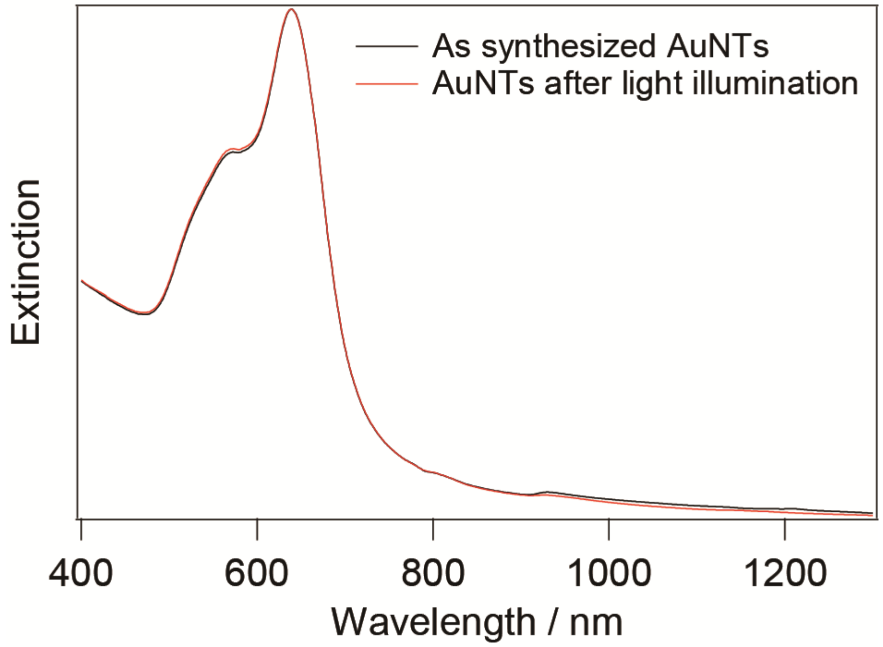

Figure S1. Optical absorption spectra of AuNTs obtained (black) before and (red) after light illumination (λ > 600 nm) for 1 h.

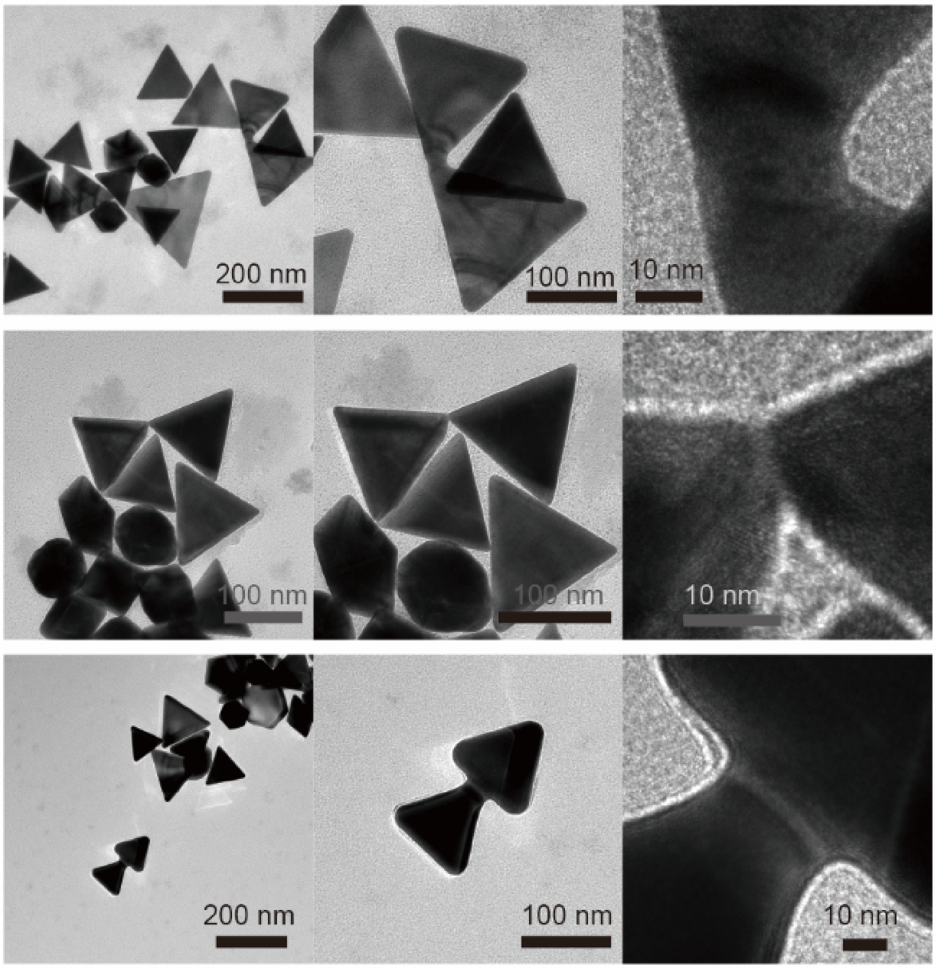

Figure S2. Transmission electron microscopy (TEM) images of welded gold nanotriangles (AuNTs) obtained after light illumination (λ > 600 nm) from Figure 1c at different magnifications.

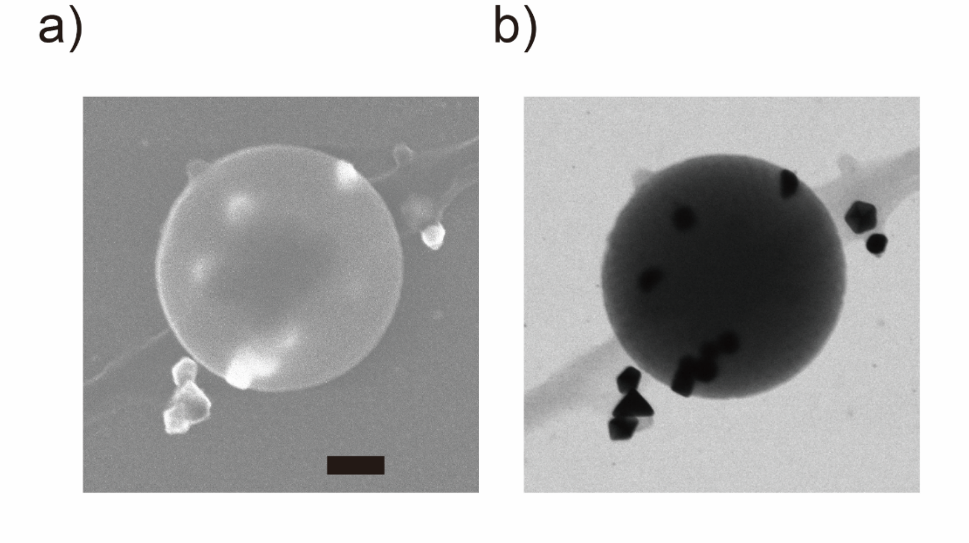

Figure S3. (a) SE and (b) bright-field STEM images of a synthesized gold NP encapsulated in silica nanocapsule (scale bar: 100 nm).

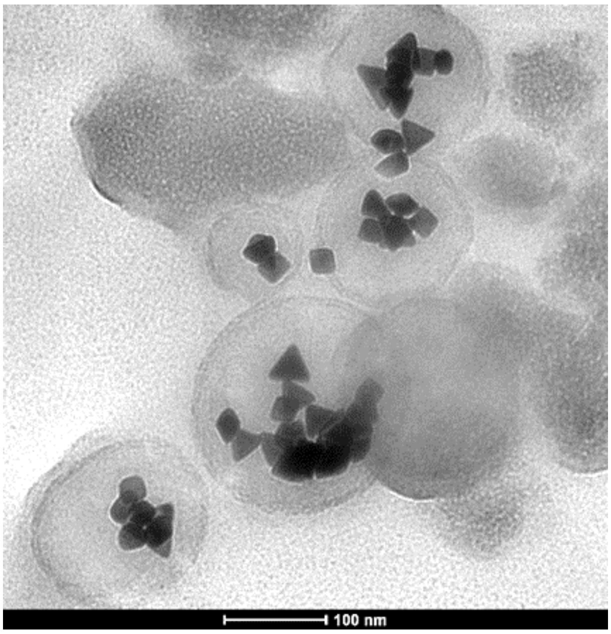

Figure S4. Representative TEM image of the encapsulated gold NPs to obtain Figure 2d.

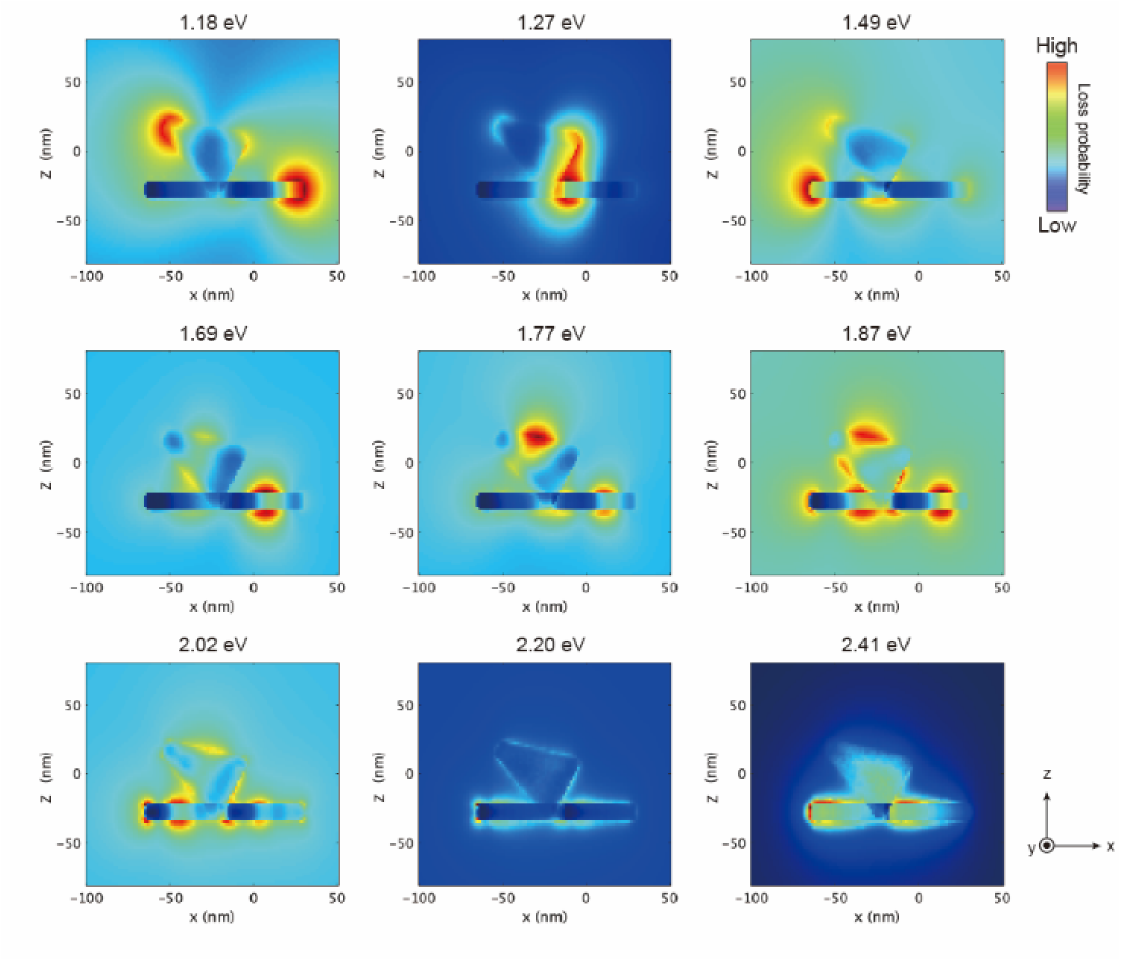

Figure S5. Simulated EEL maps of the welded gold nanostructure observed in Fig. 3a. The welding two AuNTs was placed on the xy plane, and observed from y-axis.
